# 1-Formyl-*β*-carboline Derivatives Block Newcastle Disease Virus Proliferation through Suppressing Viral Adsorption and Entry Processes

**DOI:** 10.3390/biom11111687

**Published:** 2021-11-12

**Authors:** Chongyang Wang, Ting Wang, Jiangkun Dai, Zhiyuan An, Ruochen Hu, Liuyuan Duan, Hui Chen, Xiangwei Wang, Zhili Chu, Haijin Liu, Juan Wang, Na Li, Zengqi Yang, Junru Wang

**Affiliations:** 1 College of Chemistry and Pharmacy, Northwest A&F University, Xianyang 712100, China; wangcy2019@outlook.com (C.W.); daijkun@hotmail.com (J.D.); anzhiyuan@nwafu.edu.cn (Z.A.); 2 College of Veterinary Medicine, Northwest A&F University, Xianyang 712100, China; t_wang@nwafu.edu.cn (T.W.); 18829349275@163.com (R.H.); dly190603@163.com (L.D.); chenhui.0825@outlook.com (H.C.); zhilichu@126.com (Z.C.); liuhaijin@nwafu.edu.cn (H.L.); juan.wang1234@hotmail.com (J.W.); 3 State Key Laboratory of Veterinary Etiological Biology, Lanzhou Veterinary Research Institute, Chinese Academy of Agricultural Sciences, Lanzhou 730046, China; wangxiangwei@caas.cn; 4 Instrumental Analysis Center, Xi’an Jiaotong University, Xi’an 710049, China

**Keywords:** *β*-carbolines, anti-viral activity, Newcastle disease virus, HN protein, PI3K/Akt signaling pathway

## Abstract

Newcastle disease virus (NDV) is one of the highly contagious pathogens causing devastating economic effects on the global poultry industry. In the present study, three 1-formyl-*β*-carboline derivatives (compounds **6**, **7**, and **9**) were found to be potent inhibitors of different genotypes of NDV with IC_50_ values within 10 μM, which are similar to ribavirin. The virus titers were decreased by the presence of 1-formyl-*β*-carboline derivatives in a dose-dependent manner, and the inhibition rate was found to exceed 90% at the concentration of 20 μM. These compounds mainly suppressed the adsorption and entry processes of NDV lifecycle. Through DARTS, CETSA, and RBC binding assay, these compounds were identified as novel HN inhibitors, which could directly interact with the NDV HN protein to affect the adsorption of NDV. Furthermore, they could inhibit the entry of NDV through suppressing the PI3K/Akt pathway rather than the ERK pathway. The PI3K/Akt pathway was proved to be involved in NDV entry. Our findings reveal a unique mechanism through which 1-formyl-*β*-carboline derivatives restrain NDV infection. Moreover, these compounds represent suitable scaffolds for designing novel HN inhibitors.

## 1. Introduction

The Newcastle disease (ND) is one of the most severe viral diseases in the poultry industry worldwide. ND is caused by the infection of the virulent Newcastle disease virus (NDV) belonging to the *Avulavirinae* subfamily, which has a negative-strand RNA genome that encodes at least six viral proteins including two surface glycoproteins, the fusion protein (F), and the hemagglutinin-neuraminidase protein (HN). Among these, HN is an attachment protein that is essential to cell binding [1]. Based on its virulence, NDV is classified into three types: lentogenic, mesogenic, and velogenic. The velogenic strains can cause a huge loss in commercial and backyard poultry [2]. At present, the most commonly used method of control for ND is vaccination [3].

*β*-Carboline alkaloids are distributed widely in various plant sources, mainly in Simaroibaceae, Zygophyllaceae, Rutaceae, and Caryophyllaceae [4]. These alkaloids are known to display a diverse biological activity, such as anti-cancer, anti-oxidative, anti-inflammatory, and anti-Alzheimer effects [5,6,7,8]. Recent studies have also revealed *β*-carboline alkaloids to be active against the herpes simplex virus, influenza A virus, and dengue viruses as well as enterovirus 71 and human immunodeficiency virus [9,10,11,12]. 9-Methylharmine was effective to decrease the replication of the dengue virus (DENV) with an IC_50_ value at 3.2 ± 0.6 μM [13].

Different anti-viral agents block various steps of the viral life cycle, including host receptor binding, viral entry, replication, assembly, and budding. In particular, destroying the early stage of the virus life cycle, including host receptor binding or viral entry, can effectively inhibit virus proliferation. Molecules that occupy the specific region within the binding pocket of the viral protein are developed to block the virus–cell interaction. For the paramyxoviruses, the HN protein-mediated attachment to the cell surface receptor (sialic acid receptor) is the first step. HN protein, a type II transmembrane glycoprotein that presents on the surface of virions as a tetramer, consists of two domains, including an N-terminal transmembrane domain and a C-terminal globular head domain [14]. The globular head domain contains two sialic acid binding sites, called site I and site II, which are essential for receptor binding [15]. Thus, small molecules, which can bind to sialic acid binding sites, have the potential to inhibit NDV.

After attaching to cells, viruses can enter cells through various processes. Enfuvirtide is a peptidic mimic of HR2 domain of HIV-1 gp41, which is responsible for the fusion process. Inspiringly, Enfuvirtide is the first fusion inhibitor approved by the FDA [16]. During NDV infection, the F protein is responsible for virus–cell membrane fusion, thus assisting viral entry [17,18]. Besides membrane fusion, NDV was also found to enter cells by caveolae/clathrin-mediated endocytosis [19,20]. The clathrin-mediated endocytosis could be regulated by the PI3K/Akt and ERK signalling pathways [21,22]. The PI3K/Akt signalling pathway participates in the cellular entry of different viruses. Inhibitors of PI3K significantly reduced the entry of Ebola virus through regulating vesicular trafficking [17]. Meanwhile, the entry of Vaccinia virus depends on the activation of the PI3K/Akt signalling pathway [18]. The ERK pathway, activated by HSV-1, controls early cofilin phosphorylation and cell ruffle formation to regulate the entry of HSV-1 [19].

In earlier studies, our research showed that a series of *β*-carboline derivatives, including monomers and dimers, exhibit potential anti-tumor, anti-microbial, and anti-viral activities [23,24,25]. Previous works also found that substituents at C^1^, C^3^, and C^6^ of the *β*-carboline skeleton significantly improved the anti-viral activity [13,26]. Therefore, twelve *β*-carboline derivatives with substituents at C^1^, C^3^, and C^6^ were selected for an evaluation of their in vitro anti-viral activity against NDV. Three 1-formyl-*β*-carboline derivatives were identified as novel NDV inhibitors through suppressing the adsorption and entry processes of the NDV lifecycle.

## 2. Materials and Methods

### 2.1. β-Carboline Derivative Samples, Reagents, Cell Lines, and Virus

Twelve C^1^-, C^3^-, or C^6^-modified *β*-carboline derivatives were synthesized as previously described [27,28]. The structures of these *β*-carboline derivatives are summarized in Table 1.

Dynasore, U0126, LY294002, and ribavirin were purchased from Sigma-Aldrich (St. Louis, MO, USA). All compounds were dissolved in dimethyl sulfoxide (DMSO) for in vitro studies. The protease inhibitor cocktail and the phosphatase inhibitor were obtained from TargetMol (Boston, MA, USA). Antibodies against β-actin, p-ERK, ERK, p-Akt, Akt, goat anti-rabbit IgG, and goat anti-mouse IgG were purchased from Cell Signaling Technology (Topsfield, MA, USA). The mAb against NDV nucleoprotein was prepared in our laboratory.

DF-1, Hela, Vero, and BHK-21 cells originally obtained from ATCC (Manassas, VA, USA) were purchased from the Cell Bank of Chinese Academy Sciences (Shanghai, China). These cells were cultured in Dulbecco’s modified Eagle’s medium (DMEM) with 10% fetal bovine serum (Gibco, Amarillo, TX, USA) at 37 °C with 5% CO_2_.

Four NDV strains, including F48E9, Blackbird/China/08, PPMV-1/SX-01/Ch/15, and La Sota were provided by the College of Veterinary Medicine, Northwest A&F University (Xianyang, China). These viruses were plaque-purified three times. Viruses were propagated in 9-day-old to 11-day-old SPF chicken embryos, titrated by plaque assay, and stored at –80 °C.

### 2.2. Cytotoxicity Assay

The cytotoxicity assay was performed as previously described [25]. Briefly, DF-1 cells were plated in 96-well plates and incubated with different concentrations of the various *β*-carboline derivatives in triplicates for 48 h. Next, the CCK-8 reagent (10 μL; TargetMol, Boston, MA, USA) was dispensed into each well, and the plates were incubated for an additional 2 h under the same conditions. The optical density was measured at 450 nm (Model 680 Microplate Reader; Bio-Rad, Hercules, CA, USA).

### 2.3. Plaque Assay

The plaque assay was performed as previously described [25].

### 2.4. “Time of Drug-Addition” Assay

The “time of drug-addition” assay was performed to determine the mode of activity. Briefly, DF-1 cells were infected with NDV (MOI = 0.01). The corresponding compound was added at a concentration of 20 μM at different time points before infection or post-infection: 6 h or 2 h before infection (eliminated at 0 h before NDV infection); 0 h, 2 h, 4 h, 8 h, or 12 h post-infection (remained in the medium until 24 h post-infection). At 24 h post-infection, the cell culture supernatant was collected, and the viral yield was assessed by a plaque assay.

### 2.5. Adsorption Assay

Adsorption assays were performed as reported previously, with some modifications [29]. Briefly, DF-1 cells were treated as described below. (i) DF-1 cells were cooled at 4 °C for 1 h prior to infection with pre-treatment-NDV at 4 °C for 1 h (pre-treatment of NDV: the corresponding compound (20 μM) was added into NDV with titer at 200,000 PFU/100 μL). Next, this mixture was incubated at room temperature for 2 h before diluting to 100 PFU/100 μL; alternatively: (ii) DF-1 cells were incubated with the corresponding compound (20 μM) at 37 °C for 5 h, then cooled at 4 °C for 1 h prior to infection with NDV (100 PFU) at 4 °C for another 1 h; alternatively: (iii) DF-1 cells were cooled at 4 °C for 1 h, infected with NDV (100 PFU) and simultaneously supplemented with the corresponding compound (20 μM) at 4 °C for 1 h. Unbound viruses and residual compounds were washed three times with pre-cooled PBS. Next, DF-1 cells were covered with medium-containing methylcellulose (1%). Plaques were visualized and counted by staining them with crystal violet after 72 h.

### 2.6. Entry Assay

To determine the inhibitory activity of compounds on viral entry, the entry assay was conducted. Briefly, DF-1 cells were cooled at 4 °C for 1 h prior to infection with NDV (MOI = 5) at 4 °C for another 1 h to allow for viral adsorption. Next, cells were washed three times with pre-cooled PBS to remove unbound viruses, followed by incubation with the corresponding compound at 37 °C/5% CO_2_ for 4 h. At 4 h post-incubation, cells were used for indirect immunofluorescence or harvested for Western blot.

### 2.7. Homology Modeling and Molecular Docking

The 3D structure prediction was carried out by an alignment of target sequences (Genbank Accession Number: AY034892) with template structures (PDB ID: 4FZH) with the help of the Swiss model [30,31]. UCSF chimera and Sybyl X-2.0 were used for docking studies. All the structures were drawn in Sybyl X-2.0, and the energy was optimized with the Tripos force field and by the Gasteiger–Hückel method.

### 2.8. Drug Affinity Responsive Target Stability (DARTS)

DARTS was performed as previously reported, with some modifications [32,33]. Briefly, DF-1 cells were transfected with the HN-flag. At 30 h post-transfection, cells were washed with ice-cold PBS, lysed with ice-cold M-PER reagent, and supplemented with protease and phosphatase inhibitor cocktails on ice for 10 min. Cell lysates were centrifuged at 12,000× *g* at 4 °C for 20 min, and supernatants were mixed with 10× TNC buffer. The mixtures were incubated with DMSO or the corresponding compound at 4 °C overnight before the addition of pronase (Sigma-Aldrich, St. Louis, MO, USA) at room temperature. At 10 min post-incubation, a 10× protease inhibitor cocktail was added to stop the digestion. The samples were added to a 5× SDS-PAGE loading buffer and heated at 70 °C for 10 min. Subsequently, the resulting samples were subjected to Western blot analysis.

### 2.9. Cellular Thermal Shift Assay (CETSA)

CETSA was performed as previously reported, with some modifications [34,35]. Briefly, DF-1 cells were transfected with the HN-flag. At 30 h post-transfection, cells were washed with ice-cold PBS, lysed with ice-cold M-PER reagent, and supplemented with protease and phosphatase inhibitor cocktails on ice for 10 min. The supernatants were incubated with DMSO or the corresponding compound (200 μM) at 4 °C overnight. After incubation, the samples were divided into 100 μL aliquots and heated at the indicated temperature for 5 min in a PCR machine, followed by 10 min of cooling on ice. The soluble proteins were separated by centrifugation and subjected to Western blot analysis.

### 2.10. Plasmids, Transfections, and RBC Binding Assay

The HN-flag plasmid was constructed based on the sequence of the F48E9 strain (Genbank Accession Number: MG456905). The transfection was performed according to the protocol of the Turbofect transfection reagent (Thermo Fisher, Waltham, MA, USA).

BHK-21 cells were transfected with the HN-flag or vector. At 24 h post-transfection, cells were washed and incubated with a 1% suspension of red blood cells (RBCs) supplemented with or without each compound (20 μM) at 4 °C for 1 h. Plates were softly shaken every 5 min. Next, cells were washed three times with PBS to remove unbound RBCs, lysed in 100 μL of 50 mmol/L NH_4_Cl, and centrifuged at 12,000× *g* for 5 min. The liquid phase was transferred to a 96-well plate. The percentage of binding RBCs was determined by the measurement of the absorbance at 405 nm.

### 2.11. RNA Extraction and Reverse Transcription and Quantitative Real-Time PCR (RT-qPCR)

The RNA extraction and reverse transcription and quantitative real-time PCR (RT-qPCR) were performed as previously described [25]. The primers used in this study are listed in Table 2.

### 2.12. Western Blot Analysis

Western blot analysis was performed by standard procedures as previously described [25]. The intensities of target bands were analyzed using the Image J software 1.52 v (NIH, Bethesda, MD, USA).

### 2.13. Indirect Immunofluorescence

Cells were washed three times with PBS and fixed in 4% paraformaldehyde, permeabilized with 0.5% Triton X-100 for 20 min, blocked in PBST containing 5% BSA at 37 °C for 1 h, and then incubated with anti-NP Ab at 37 °C for 1 h. Next, cells were incubated with Alexa Fluor 488-conjugated goat anti-mouse IgG. Actin filaments were stained with TRITC-phalloidin (2 μg/mL) at 37 °C for 0.5 h. Cell nuclei were stained with DAPI (1 μg/mL) for 10 min. Cells were visualized using a confocal laser-scanning microscope (Revolution WD, Andor, Pittsfield, MA, USA).

### 2.14. Statistical Analysis

All treatments were applied in triplicates, and each experiment was independently repeated at least three times. Results are presented as means ± standard deviations of the mean. The values of CC_50_ or IC_50_ were calculated using the GraphPad Prism 6.0 software (USA). The statistical analysis was done by a two-tailed Student’s *t*-test using the GraphPad Prism 6.0 software. Statistical significance: ns (not significant), * *p* < 0.05, ** *p* < 0.01, and *** *p* < 0.001.

## 3. Results

### 3.1. Cytotoxic and Anti-Viral Activity of β-Carbolines In Vitro

The anti-NDV activity of twelve *β*-carboline derivatives with substituents at C^1^, C^3^, and C^6^ was studied. The cytotoxicity of all compounds in DF-1 cells is shown in Figure S1. The anti-viral activity assays were performed with the corresponding compound at up to 20 μM, except for compounds **8** (which were the most cytotoxic) indicating that the C^3^ methoxycarbonyl group adversely affects the cell viability.

**Figure 1:**
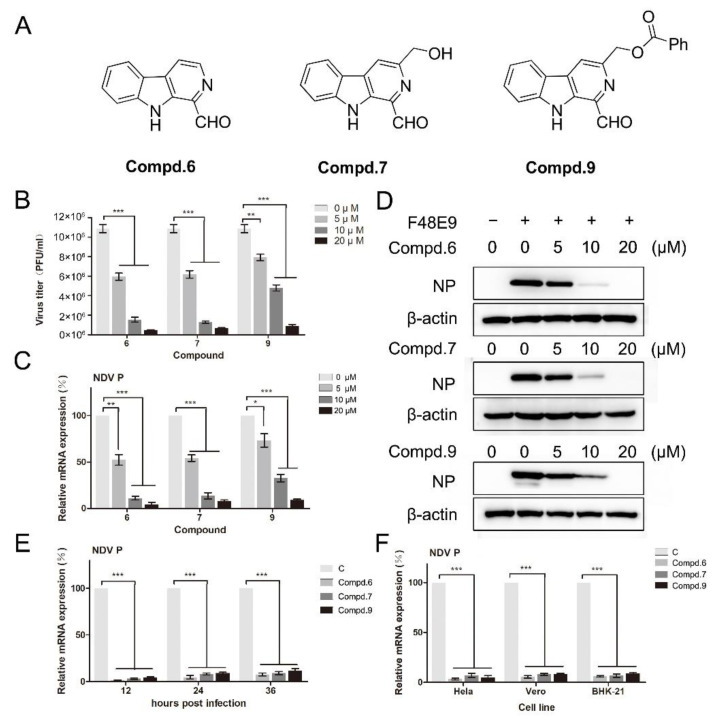
1-formyl-*β*-carboline derivatives inhibit the NDV proliferation in a dose-dependent manner. (**A**) Molecular structures of compounds **6**, **7**, and **9**. (**B**–**D**) DF-1 cells were infected with F48E9 (MOI = 0.01). After adsorption (1 h), cells were covered with DMEM containing different concentrations of each compound. At 24 h post-infection, the virus yield in the supernatant was measured by plaque assay (**B**). The relative mRNA expression was measured by RT-qPCR. (**C**). Protein levels were measured by Western blot (**D**). (**E**) DF-1 cells, infected with F48E9 (MOI = 0.01), were treated with each compound (20 μM). At the indicated time point, cells were harvested to determine the viral mRNA expression by RT-qPCR. (**F**) Hela, Vero, and BHK-21 cells were infected with F48E9 (MOI = 0.01) and treated with each compound (20 μM), respectively. At 24 h post-infection, cells were harvested to determine the viral mRNA expression by RT-qPCR. The data represent the mean ± SD of three independent experiments. The statistical significance was analyzed using a two-tailed Student’s *t*-test: * *p* < 0.05; ** *p* < 0.01; *** *p* < 0.001.

Based on the results of cell viability, the anti-viral activity against NDV was screened by plaque assay and RT-qPCR at the concentration without cytotoxicity. DF-1 cells, infected with F48E9, were supplemented with the corresponding compound for 24 h. Plaque-forming units were calculated. Preliminary results showed that compounds **6**, **7**, and **9** had inhibitory activity against F48E9 in a dose-dependent manner. In the presence of 20 μM of compound **6**, the virus titer was decreased by 95%. The treatment with compounds **7** and **9** (20 μM) caused a significant reduction of 93% and 91%, respectively (Figure 1B). The other compounds did not show any effect on the NDV proliferation (Figure S2). Meanwhile, the inhibitory effect of these compounds was confirmed at the mRNA and protein levels (Figure 1C,D). These compounds also exert the inhibitiory effects until 36 h post-infection (Figure 1E). Moreover, these compounds showed an excellent anti-NDV effect in various cell lines (Figure 1F).

The CC_50_ and IC_50_ values of the three potential inhibitors were determined by CCK-8 and plaque assays, respectively. The selectivity index (SI) was calculated as the ratio of CC_50_/IC_50_. Compound **6** exhibited the best SI value (8.9). The IC_50_ value of compound **6** was determined to be 7.5 μM. Based on the IC_50_ values, the anti-NDV activity of these compounds was in the order of **6** > **7** > **9**. Ribavirin, a well-known anti-viral agent, was used as a positive control. These compounds showed a similar anti-viral activity compared with ribavirin (Table 3).

The anti-viral activity of the various compounds against other NDV strains was analyzed by plaque assay. The results showed they could inhibit diverse genotypes of NDV displaying similar anti-viral effects against all strains (Table 4). These results indicate that the anti-NDV activity of these compounds was not strain-specific. Overall, these results highlighted the excellent anti-NDV activity of compounds **6**, **7**, and **9**.

### 3.2. 1-Formyl-β-Carboline Derivatives Disrupt the Early Stages of NDV Life Cycle

**Figure 2:**
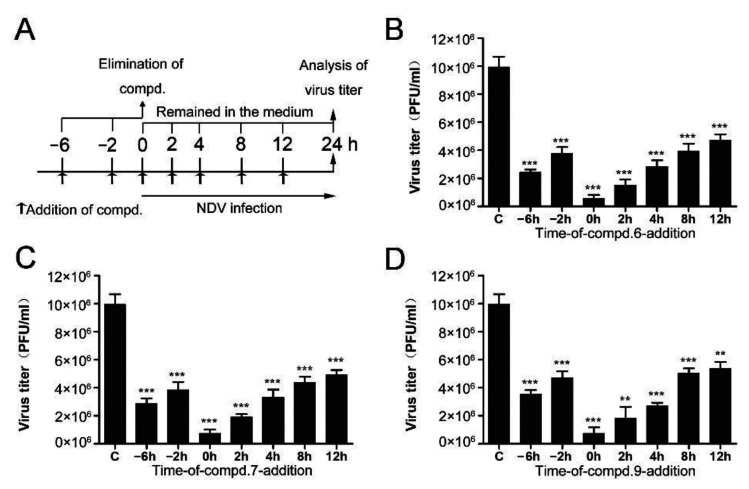
Time of drug-addition assay. (**A**) Compound treatment and NDV infection schemes in the “time of drug-addition” assay. (**B**–**D**) The virus yield in the supernatant was measured by plaque assay at 24 h post-infection. The data represent the mean ± SD of three independent experiments. The statistical significance was analyzed using a two-tailed Student’s *t*-test: ns (not significant); ** *p* < 0.01; *** *p* < 0.001.

In order to clarify which stage of the NDV lifecycle is targeted by these compounds, the “time of drug-addition” assay was performed. Compounds were added at different time points and supernatants were collected at 24 h post-infection. Compound treatment and NDV infection schemes in the “time of drug-addition” assay are shown in Figure 2A. The results summarized in Figure 2 revealed that these compounds both showed anti-viral effects when they were added before or post NDV infection. When compounds were added after the infection of NDV, they significantly decreased the production of NDV virions, especially at the early stage (Figure 2). When cells were treated with compounds at 0 h post-infection, three compounds exhibited superior activity, with an inhibitory rate higher than 90%. When cells were treated with compounds at 4 h post-infection, **6**, **7**, and **9** exhibited lower inhibitory rates (71%, 66%, and 72%, respectively). As we can see, the inhibitory rates were gradually decreased as time prolonged. These results indicated that these compounds mainly affect the early stages of NDV proliferation.

### 3.3. 1-Formyl-β-Carboline Derivatives Inhibit NDV Adsorption through Interacting with HN

**Figure 3:**
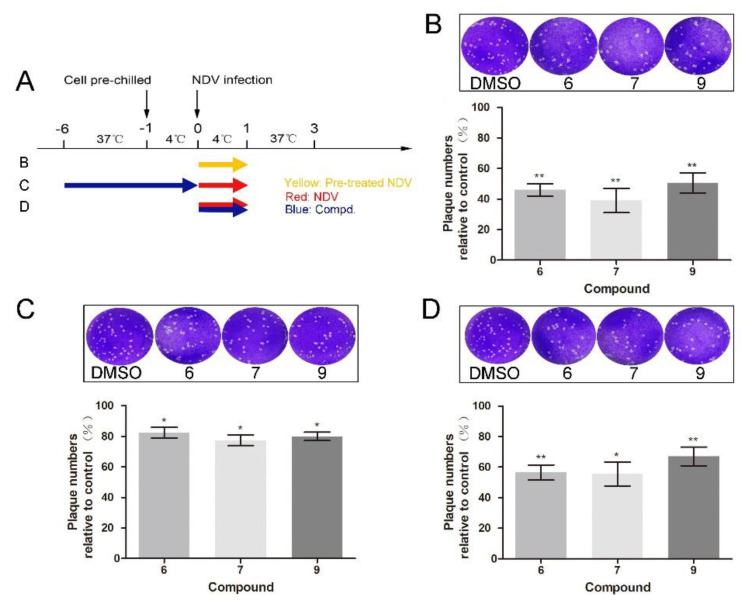
1-Formyl-*β*-carboline derivatives inhibit NDV adsorption. (**A**) Compound treatment and NDV infection schemes in the adsorption assay. (**B**–**D**) After infection with pre-treated or untreated NDV (100 PFU), DF-1 cells were covered with a medium containing methylcellulose. At 72 h post-infection, the virus yield was analyzed. Dynasore (100 μM) was used as a positive control. The data represent the mean ± SD of three independent experiments. The statistical significance was analyzed using a two-tailed Student’s *t*-test: * *p* < 0.05; ** *p* < 0.01.

To further dissect which stage was targeted by 1-formyl-*β*-carboline derivatives, adsorption assays were performed. Compound treatment and NDV infection schemes in the adsorption assay are displayed in Figure 3A. As shown in Figure 3B, when the NDV was pre-incubated with compounds **6**, **7**, or **9** at room temperature for 2 h, the virus-binding to DF-1 cells significantly decreased. Infectious virions were decreased by 54% after treatment with compound **6**. Meanwhile, treatment with compounds **7** or **9** decreased the virus-binding to cells by 61% or 49%, respectively (Figure 3B). While treated with compounds **1** or **5**, NDV virions binding to cells did not decrease (Figure S3A). However, cells pre-treated with the corresponding compound only caused a slight decrease (<25%; Figure 3C). When compound **6** was added to the NDV, a decrease of about 43% was observed. Treatment with compounds **7** or **9** also significantly decreased the virions by 44% and 33%, respectively (Figure 3D). Overall, these results suggested that all three compounds inhibit NDV adsorption.

**Figure 4:**
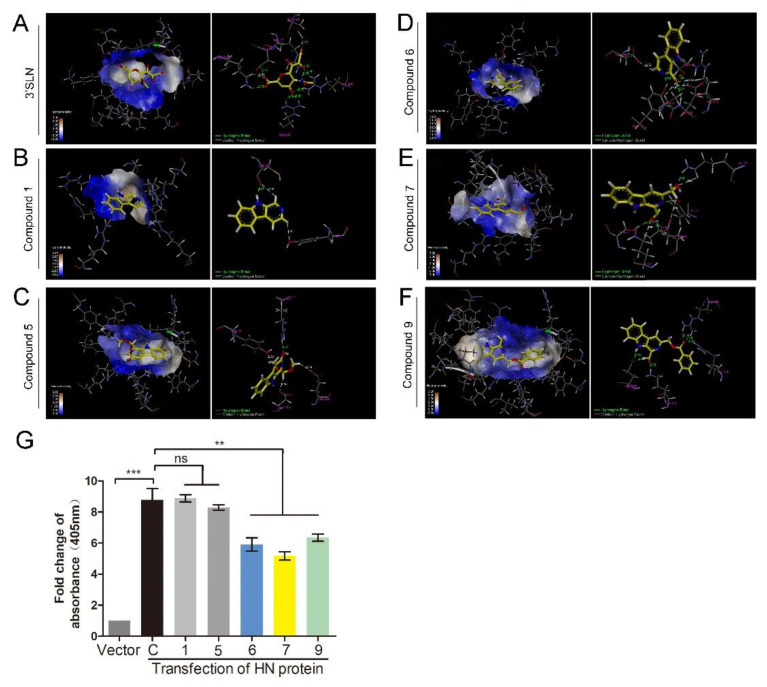
1-formyl-*β*-carboline derivatives interact with the HN protein. (**A**–**F**) Docking results of 3′SLN (**A**), compounds **1** (**B**), **5** (**C**), **6** (**D**), **7** (**E**), and **9** (**F**) in the binding site of the HN protein. Hydrogen bonds are shown as dotted lines, with a distance unit of Å. (**G**) BHK-21 cells were transfected with HN or vector. At 24 h post-transfection, cells were washed and incubated with a 1% suspension of red blood cells (RBCs), supplemented with or without each compound (20 μM), at 4 °C for 1 h. Next, the absorbance was measured at 405 nm. The statistical significance was analyzed using a two-tailed Student’s *t*-test: ns (not significant); ** *p* < 0.01; *** *p* < 0.001.

The HN protein is involved in NDV adsorption. Therefore, a molecular docking study was performed to predict whether these compounds could interact with the HN protein. The 3D structure of the HN protein of F48E9 was carried out by a target–template alignment, using a Swiss Model Server. The structure of HN (PDB: 4FZH) of an NDV Ulster strain was used as the template [15]. The sequence similarity of the target protein (F48E9) with the template protein (Ulster strain) was found to be 95.98%. Following the construction of the F48E9 HN model, molecular docking was conducted to study the interaction between the HN protein and each of the compounds. The avian receptor analog (3′-sialyl-N-acetyllactosamine, 3′SLN) was used as a positive control, while compounds **1** and **5** were used as negative controls. According to the in silico assay, as expected, 3′SLN exhibited the best binding affinity with a score of 8.0508 and multiple hydrogen bonds were formed with the Arg 174, Arg 416, Arg 498, Lys 236, etc. (Figure 4A). Compound **1** and **5** hardly bound to HN with a score of 3.3810 and 4.1407, respectively (Figure 4B,C). Compound **9** exhibited the best total score among three active compounds (7.3323), while compounds **6** and **7** displayed a score of 5.4408 and 6.1359, respectively. All compounds were selected to dock into the catalytic site of the HN protein. Figure 4D highlights the interaction of **6** with the Ser 418 and Tyr 317 residues (hydrogen bonding). The hydrogen bond distances were calculated as 1.990 and 1.890 Å, respectively. The interaction between **7** and HN is shown in Figure 4E. The oxygen atom of the formyl group at C^1^ forms two hydrogen bonds (2.01 Å and 2.43 Å) and two carbon–hydrogen bonds (2.53 Å and 2.86 Å) with the amino acids of the HN protein. As shown in Figure 4F, three hydrogen bonds (1.92 Å, 2.16 Å, and 2.35 Å) were formed between compound **9**, Arg 498, and Arg 174. The oxygen atom of the formyl group at C^1^ is adjacent to the Lys 236 residue of the target, thus forming a hydrogen bond (1.95 Å). These results suggested that compounds **6**, **7**, and **9** are likely to strongly bind to the HN protein. To confirm the results of the molecular docking study, the RBCs binding assay was conducted. As shown in Figure 4G, transfection with HN protein significantly increased the amounts of RBCs adsorbed on the cell surface, while incubation with three active compounds could significantly decrease the amounts of RBCs adsorbed on the cell surface. Meanwhile, compounds **1** and **5** showed no effect on RBCs binding to cells. These results confirm that compounds **6**, **7**, and **9** could interfere with the interaction between HN and RBCs.

**Figure 5:**
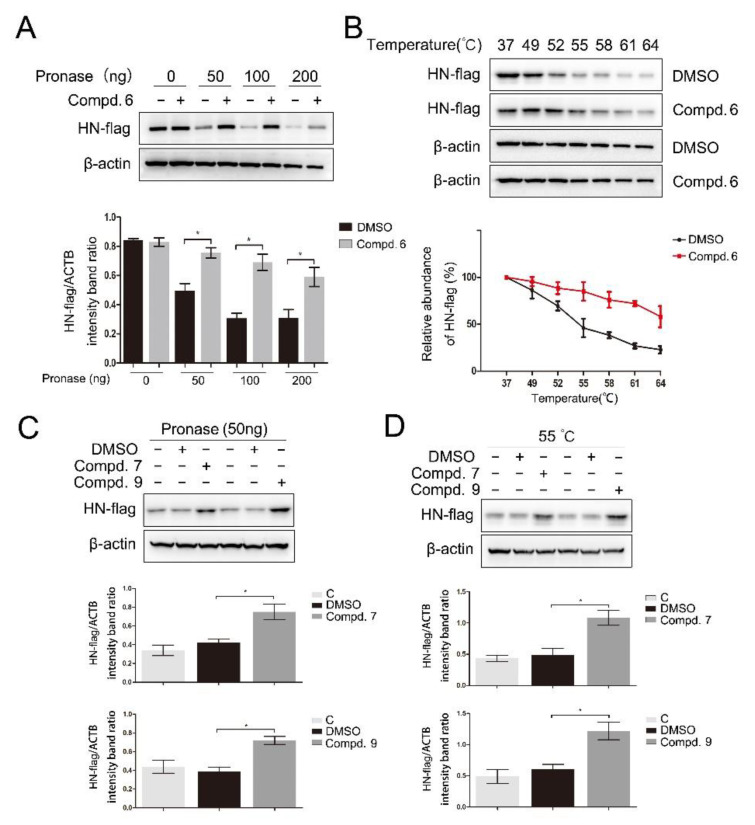
Direct binding of 1-formyl-*β*-carboline derivatives with the HN protein. DF-1 cells, transfected with the HN-flag, were lysed with M-PER lysis buffer and incubated with 1-formyl-*β*-carboline derivatives (200 μM) or DMSO. (**A**) The cell lysates were treated with the indicated concentration of pronase and protein was measured by Western blot (up). The HN-flag protein levels relative to ACTB levels were determined by densitometry (down). (**B**) The cell lysates were heated at the indicated temperature and protein was measured by Western blot (up). The HN-flag protein levels relative to ACTB levels were determined by densitometry (down). (**C**) The cell lysates were treated with pronase (50 ng) and protein was measured by Western blot (up). The HN-flag protein levels relative to ACTB levels were determined by densitometry (down). (**D**) The cell lysates were heated at 55 °C and protein was measured by Western blot (up). The HN-flag protein levels relative to ACTB levels were determined by densitometry (down). The data represent the mean ± SD of three independent experiments. The statistical significance was analyzed using a two-tailed Student’s *t*-test: * *p* < 0.05.

To further validate the direct interaction between the selected compounds and HN, drug affinity responsive target stability (DARTS) and cellular thermal shift assay (CETSA) were performed. As shown in Figure 5A, compound **6** was found to protect the HN-flag from the proteolytic enzyme at various concentrations of pronase. As expected, compound **6** also increased the thermal stability of the HN-flag (Figure 5B). Meanwhile, treatment with **7** and **9** increased the protein stability in the presence of pronase (Figure 5C) and under heating (Figure 5D), respectively. Compounds **1** and **5** were used as negative controls in DARTS and CETSA. As shown in Figure S4, two compounds protect the HN protein from neither pronase nor heating. Taken together, these results suggested that compounds **6**, **7**, and **9** directly interacted with the HN protein. These results supported that all three compounds could directly interact with the HN protein, thereby interfering with the interaction between HN and receptors.

### 3.4. 1-Formyl-β-Carboline Derivatives Inhibited NDV Entry through Suppressing the PI3K/Akt Pathway

**Figure 6:**
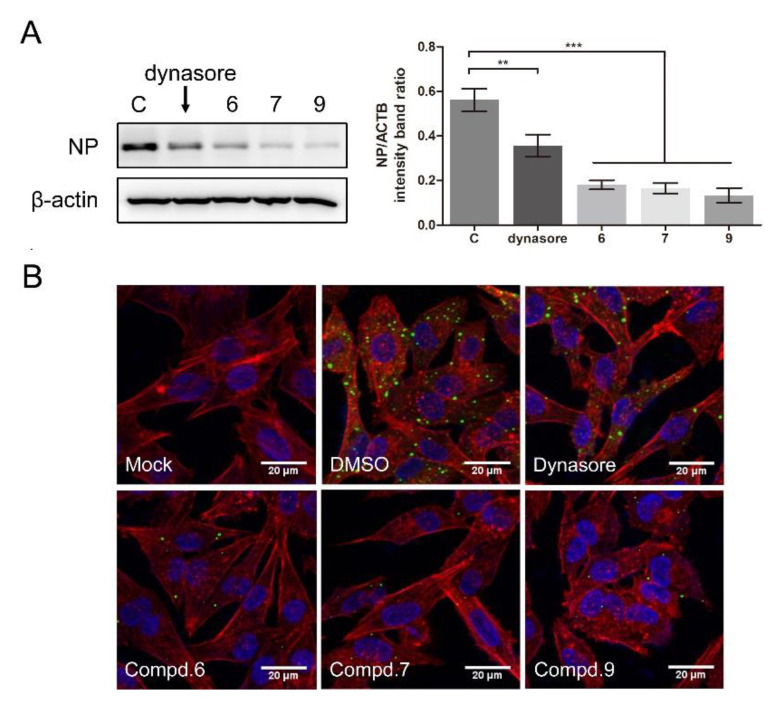
1-Formyl-*β*-carboline derivatives inhibit NDV entry. (**A**,**B**) DF-1 cells were infected with F48E9 (MOI = 5) at 4 °C for 1 h. Unbound viruses were washed away by pre-chilled PBS. Next, cells were supplemented with different medium-containing compounds (dynasore: 100 μM; compounds 6/7/9: 20 μM). At 4 h post-infection, the viral protein was measured by Western blot (**A**: left panel). The NP protein levels relative to ACTB levels were determined by densitometry (**A**: right panel). The entry of virus was measured by indirect immunofluorescence (**B**). The statistical significance was analyzed using a two-tailed Student’s *t*-test: ** *p* < 0.01; *** *p* < 0.001.

To further confirm whether the compounds interfered with NDV entry, dynasore was used as a positive control. Dynasore treatment at 100 μM did not affect cell viability (Figure S5A). When the cells were treated with dynasore or indicated compounds, the viral protein significantly decreased (0.56 ± 0.04, 0.35 ± 0.04, 0.18 ± 0.02, 0.16 ± 0.01, 0.13 ± 0.03 for DMSO, dynasore, **6**, **7**, and **9** treatment samples, respectively) (Figure 6A). As shown in Figure 6B, green fluorescence was significantly reduced in the presence of each compound, confirming that these compounds effectively block the entry of NDV, while treatment with compounds **1** and **5** had no effect on NDV entry (Figure S3B).

**Figure 7:**
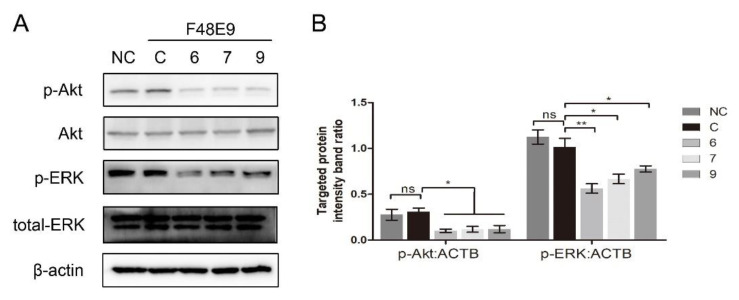
1-Formyl-*β*-carboline derivatives inhibit the PI3K/Akt and ERK pathways. (**A**) DF-1 cells, infected with F48E9 (MOI = 5), were incubated with each compound (20 μM). Next, cells were harvested to detect the Akt, p-Akt, ERK, and p-ERK at 2 h post-infection. (**B**) The intensities of the targeted protein band were normalized to ACTB, determined by densitometry. The statistical significance was analyzed using a two-tailed Student’s *t*-test: ns (not significant); * *p* < 0.05; ** *p* < 0.01.

Several *β*-carboline derivatives were reported to suppress the PI3K/Akt pathway and the ERK pathway, and those two pathways were involved in virus entry. To illustrate the mechanism of 1-formyl-*β*-carboline derivatives inhibiting NDV entry, the following assays were performed. As shown in Figure 7, the expression of p-Akt and p-ERK following the NDV entry process at 2 h post-infection was unchanged. Meanwhile, all compounds significantly decreased the expression level of p-Akt and p-ERK.

**Figure 8:**
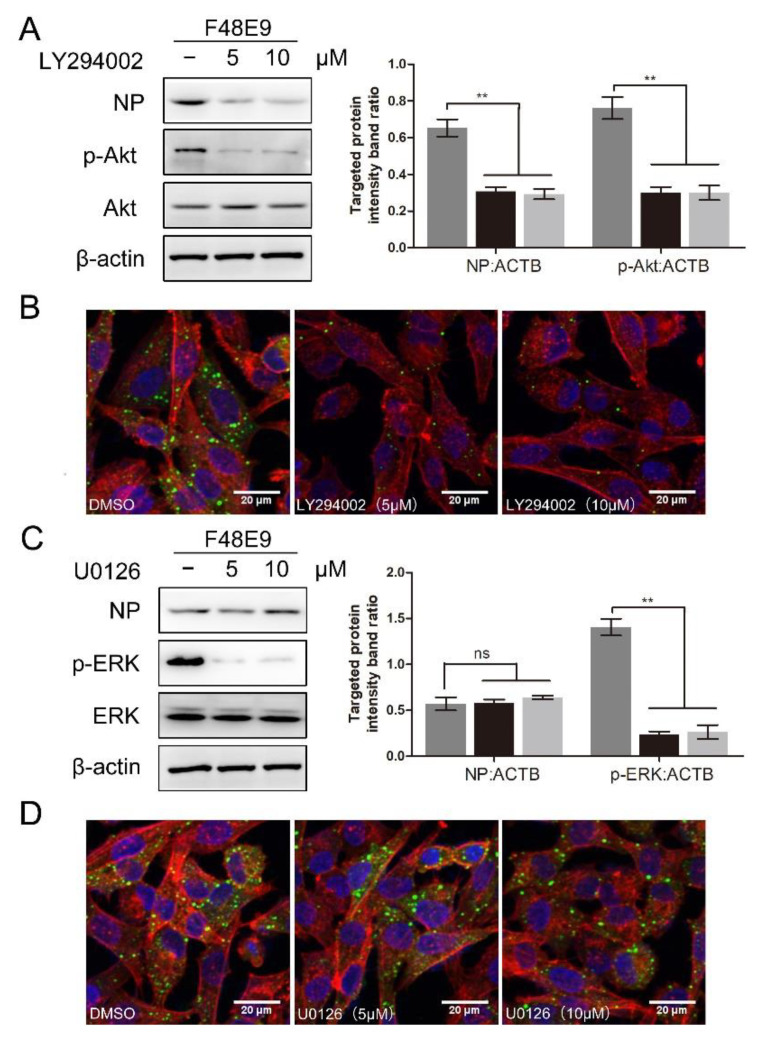
The effect of inhibition of PI3K/Akt or ERK pathway on NDV entry. (**A**,**B**) DF-1 cells were infected with F48E9 (MOI = 5) at 4 °C for 1 h. Unbound viruses were washed away by pre-chilled PBS. Next, cells were supplemented with different medium-containing LY294002. At 4 h post-infection, the viral protein was measured by Western blot (**A**: left panel). The targeted protein levels relative to ACTB levels were determined by densitometry (**A**: right panel). The entry of virus was measured by indirect immunofluorescence (**B**). (**C**,**D**) DF-1 cells were infected with F48E9 (MOI = 5) at 4 °C for 1 h. Unbound viruses were washed away by pre-chilled PBS. Next, cells were supplemented with different medium-containing U0126. At 4 h post-infection, the viral protein was measured by Western blot (**C**: left panel). The targeted protein levels relative to ACTB levels were determined by densitometry (**C**: right panel). The entry of virus was measured by indirect immunofluorescence (**D**). The statistical significance was analyzed using a two-tailed Student’s *t*-test: ns (not significant); ** *p* < 0.01.

To determine whether the PI3K/Akt or ERK pathway was involved in the NDV entry, the entry assay was performed using LY294002 or U0126. LY294002 treatment at 10 μM and U0126 treatment at 20 μM did not affect cell viability (Figure S5B,C). LY294002 treatment significantly suppressed the expression of p-Akt (0.76 ± 0.04, 0.30 ± 0.03, and 0.29 ± 0.04, respectively) and blocked the entry of NDV (0.65 ± 0.03, 0.31 ± 0.02, and 0.27 ± 0.03, respectively) (Figure 8A,B). Consistent results were observed in BHK-21 cells. As shown in Figure S6, LY294002 treatment also significantly reduced the entry of NDV in BHK-21 cells (0.62 ± 0.03, 0.30 ± 0.02, and 0.22 ± 0.02, respectively). U0126 treatment significantly suppressed the expression of p-ERK (1.41 ± 0.06, 0.23 ± 0.03, and 0.26 ± 0.07, respectively) while it did not affect the entry of NDV (0.56 ± 0.05, 0.57 ± 0.05, and 0.60 ± 0.02, respectively) (Figure 8C,D). Overall, we conclude that the PI3K/Akt pathway is involved in the NDV entry rather than the ERK pathway. 1-formyl-*β*-carboline derivatives could interfere with the NDV entry via suppressing the PI3K/Akt pathway.

## 4. Discussion

In the previous study, we found that canthin-6-one analogs could inhibit NDV proliferation along with low cytotoxicity [25]. Thus, we further studied the anti-NDV activity of a series of *β*-carboline derivatives in this research. Compounds **6**, **7,** and **9** showed strong inhibition ability against different NDV strains with better anti-viral activity compared with the canthin-6-one analogs.

A variety of *β*-carboline derivatives were reported to show different modes of action in viral infections. Several 1,3-disubstituted *β*-carboline derivatives displayed anti-viral activity against HSV-1 before and during virus adsorption [36]. 9-Methylharmine exerted an inhibitory effect on DENV-2 through impairing the maturation and release of virus particles [15]. 9-Methylnorharmane, 9-methylharmane, and 6-methoxyharmane had no effect on virus adsorption or entry, but interfered with the late-stage viral infection [37]. Unlike all these reported compounds, in this study, 1-formyl-*β*-carboline derivatives exhibited an anti-NDV activity in the early stage of the NDV infection, indicating novel mechanisms underlying the anti-viral effect.

Several experimental approaches used here demonstrated that three compounds interfered with the NDV adsorption. The HN protein is an important surface glycoprotein displaying three functions: (i) receptor recognition of sialic acid; (ii) neuraminidase (NA) activity; and (iii) interaction with the F protein to promote fusion [38,39,40]. The recognition of the receptor is crucial to the NDV-binding activity. Ser 418, Arg 416, Tyr 317, Arg 174, Glu 258, and Lys 236 are all important residues in the HN protein, which are required for sialic acid-binding and the NA activity [41]. As can be seen in Figure 4, the formyl groups of compounds **6** and **7** were predicted to interact with several amino acid residues including Ala 400, Glu 401, Arg 416, and Ser 418. Likewise, the formyl group of **9** was predicted to interact with the Lys 236 residue. These data suggested that the formyl group is a key substituent to interfere with NDV adsorption. Furthermore, the formyl group is vital to the anti-viral activity against NDV. 1-formyl-*β*-carboline-3-carboxylic acid methyl ester was found to inhibit HIV proliferation (IC_50_ = 2.9 μM). While the formyl group was substituted by a methyl group, the anti-viral activity was found to be significantly decreased [42]. These results highlighted the importance of the formyl group in the anti-viral activity. Meanwhile, it is well-known that the influenza virus also binds to sialic acid receptors. Thus, we assume that these compounds may also interfere with the adsorption of the influenza virus.

After virus adsorption, the virus will continue to enter the host cells. Three effective compounds in this study also interfered with the NDV entry. The process of NDV entry into DF-1 cells relied on multiple strategies including clathrin-mediated endocytosis (CME) [20]. The PI3K/Akt pathway could upregulate clathrin and promote the entry of bovine ephemeral fever virus [21]. Another well-studied link between clathrin-mediated endocytosis and signaling was demonstrated in the pathway by which RTKs activate ERK [22]. Nevertheless, the role of the PI3K/Akt and ERK pathways in regulating the entry of NDV remains unknown until now. Our results demonstrated that the PI3K/Akt pathway plays an important role in the entry of NDV. An earlier report showed that the ERK pathway, activated by the Sendai virus (another member of paramyxoviruses), controls ezrin-mediated cytoskeletal rearrangements to promote viral fusion [43]. On the contrary, the entry process of Junín and Tacaribe viruses was not affected by ERK [44]. These studies indicated that the ERK pathway may exert different functions in diverse cell lines or viruses. Our results demonstrated that the ERK pathway has no effect on NDV entry into DF-1 cells. To the best of our knowledge, none of the *β*-carboline derivatives has been known to inhibit virus entry. These results expanded the anti-viral mechanism of *β*-carboline derivatives. Furthermore, comparing the inhibitiory effect of active compounds on NDV adsorption and entry, we suggest that these compounds mainly block NDV through the inhibition of NDV entry.

In summary, three 1-formyl-*β*-carboline derivatives (compounds **6**, **7**, and **9**) were found to exhibit strong inhibitory activity against diverse NDV strains through interfering with the early stages of the NDV life cycle. These compounds were proved to be novel HN inhibitors, thus interfering with NDV adsorption processes. Our data suggest that these *β*-carboline derivatives may decrease the entry of NDV via suppressing the PI3K/Akt pathway. Overall, these data illustrate the mechanism underlying the anti-NDV activity of 1-formyl-*β*-carboline derivatives.

## Figures and Tables

**Table 1:**
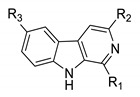
*β*-Carboline derivatives used in this study.

Compd.	R_1_	R_2_	R_3_
**1**	H	H	H
**2**	CH(OCH_3_)_2_	H	H
**3**	CH(OCH_3_)_2_	H	OCH_3_
**4**	CH(OCH_3_)_2_	COOCH_3_	H
**5**	CH(OCH_3_)_2_	OH	H
**6**	CHO	H	H
**7**	CHO	OH	H
**8**	CHO	COOCH_3_	H
**9**	CHO	CH_2_OCOPh	H
**10**	CH_2_NHBn	COOCH_3_	H
**11**	(CH_2_)_3_OH	H	H
**12**	(CH_2_)_3_OH	CH_2_OH	H

**Table 2:** Primer pairs used in this study.

RNA Target	Forward Primer (5′–3′)	Reverse Primer (5′–3′)
NDV P	TCATGCCCAGCTACCTGTCG	CTGTTGGATTTCAGACCGCATC
β-actin (DF-1)	TGGTGATGAAGCCCAGAGCAA	TGGCTTTGGGGTTCAGGGGAG
β-actin (Hela)	TGGACATCCGCAAAGACCTGT	GGAGTACTTGCGCTCAGGAGG
β-actin (BHK-21)	GAGAAGATCTGGCACCACACC	TACGACCAGAGGCATACAGGGA
β-actin (Vero)	GCGCGGCTACAGCTTCACCAC	GCCATCTCCTGCTCGAAGTCCA

**Table 3:** Cytotoxicity and anti-NDV activity of 1-formyl-*β*-carboline derivatives.

Compd.	CC_50_^a^ (μM)	IC_50_^b^ (μM)	SI ^c^
**6**	67.1 ± 6.2	7.5 ± 1.8	8.9
**7**	51.7 ± 4.5	8.2 ± 1.9	6.3
**9**	82.9 ± 7.3	10.7 ± 2.3	7.7
Ribavirin	– ^d^	8.6 ± 1.4	– ^d^

^a^ DF-1 cells were treated with different concentrations of each compound at 37 °C for 48 h. The cell viability was determined by a CCK-8 kit, and CC_50_ values were calculated (mean ± SD). ^b^ DF-1 cells, infected with F48E9 (MOI = 0.01), were treated with different concentrations of each compound for 24 h. Supernatants were harvested to determine the virus production by a plaque assay. IC_50_ values were calculated (mean ± SD). ^c^ Selectivity index; SI = CC_50_/IC_50_. ^d^ Not tested.

**Table 4:** IC_50_ values (μM) of 1-formyl-*β*-carboline derivatives against three genotypes of NDV.

Compd.	Blackbird/China/08 (IX)	PPMV-1/SX-01/Ch/15 (VI)	La Sota (II)
**6**	7.3 ± 1.2	9.4 ± 1.8	8.1 ± 1.3
**7**	12.8 ± 1.1	10.2 ± 1.3	8.5 ± 1.6
**9**	13.9 ± 2.5	15.7 ± 1.4	12.2 ± 3.4
Ribavirin	10.4 ± 2.8	11.6 ± 2.1	7.9 ± 1.5

DF-1 cells, infected with different genotypes of NDV (MOI = 0.01), were treated with different concentrations of each compound for 24 h. Supernatants were harvested to determine the virus production by a plaque assay. IC_50_ values were calculated (mean ± SD).

## Supplementary Materials

The following are available online at https://www.mdpi.com/article/10.3390/biom11111687/s1, Figure S1: Cell viability of DF-1 cells treated with *β*-carboline derivatives, Figure S2: Anti-viral activity of *β*-carboline derivatives against NDV, Figure S3: Compound **1** and **5** did not inhibit NDV adsorption and entry, Figure S4: Compound **1** and **5** had no effect on HN stability, Figure S5: Cell viability of DF-1 cells treated with inhibitors, Figure S6: LY294002 inhibits NDV entry into BHK-21 cells.
