# Supplementary material for: 1-Formyl-β-carboline Derivatives Block Newcastle Disease Virus Proliferation through Suppressing Viral Adsorption and Entry Processes
Source: Biomolecules. 2021 Nov 12;11(11):1687. doi: 10.3390/biom11111687 (PMC8616010; doi:10.3390/biom11111687)
Supplement: Supplementary file 1 [file biomolecules-11-01687-s001.zip › biomolecules-1416057-supplementary.pdf]

Supplementary data

1-Formyl-β-Carboline Derivatives Block Newcastle Disease Virus Proliferation Through Suppressing Viral Adsorption and Entry Processes

Chongyang Wang[1,†], Ting Wang[2,†], Jiangkun Dai[1], Zhiyuan An[1], Ruochen Hu[2], Liuyuan Duan[2], Hui Chen[2], Xiangwei Wang[3], Zhili Chu[2], Haijin Liu[2], Juan Wang[2], Na Li[4,*], Zengqi Yang[2,*] and Junru Wang[1,*]

[1] College of Chemistry and Pharmacy, Northwest A&F University, Yangling 712100, China;

[2] College of Veterinary Medicine, Northwest A&F University, Yangling 712100, China;

[3] State Key Laboratory of Veterinary Etiological Biology, Lanzhou Veterinary Research Institute, Chinese Academy of Agricultural Sciences, Lanzhou 730000, China;

[4] Instrumental Analysis Center, Xi'an Jiaotong University, Xi’an 710000, China;

[†] These authors contributed equally to this work.

* Correspondence: lina2021@xjtu.edu.cn (N. L); yzq1106@nwsuaf.edu.cn (Z.-Q. Y); wangjunru@nwsuaf.edu.cn (J.-R.W);
Tel.: +86-29-8709-2662 (J.-R.W)

Figure S1 displays the cytotoxicity of all β-carboline derivatives used in this study.

Figure S2 provides the effect of non-effective β-carboline derivatives on the NDV proliferation.

Figure S3 displays the effect of compounds **1** and **5** on the adsorption and entry of NDV.

Figure S4 provides the effect of compounds **1** and **5** on the stability of HN.

Figure S5 displays the cytotoxicity of inhibitors used in this study.

Figure S6 displays the effect of LY294002 on the entry of NDV in BHK-21 cells.

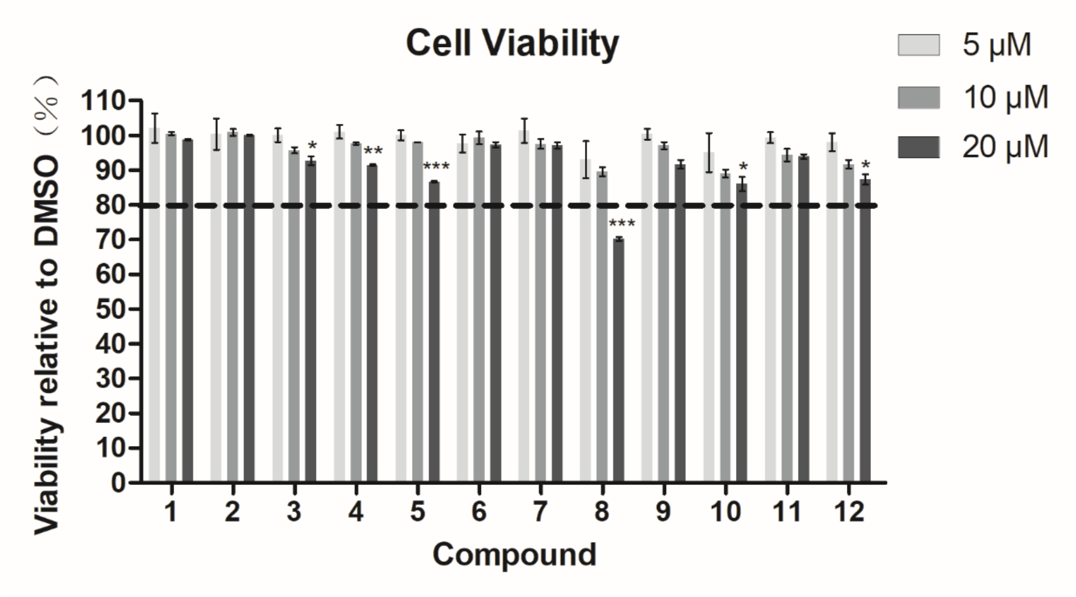

Figure S1. Cell viability of DF-1 cells treated with β-carboline derivatives.
DF-1 cells were incubated with different concentrations of each compound. At 48 h post-incubation, the cell viability was determined using a CCK-8 kit.

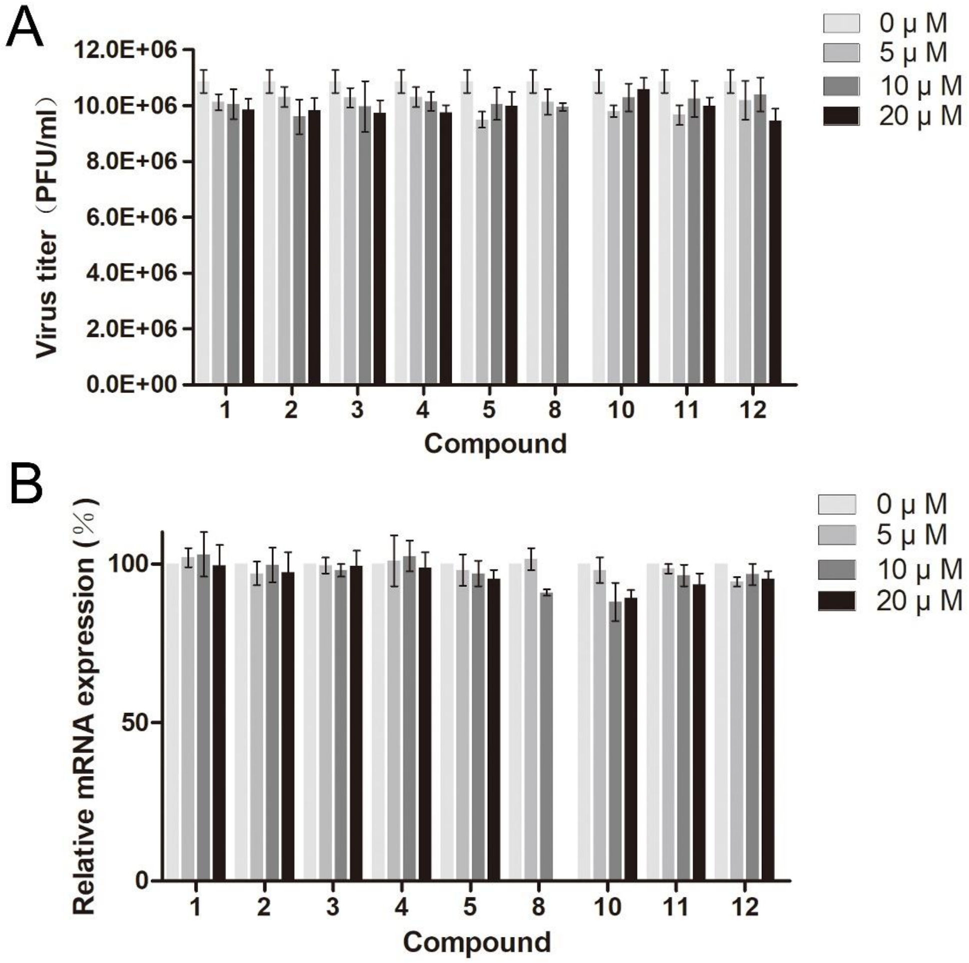

Figure S2. Anti-viral activity of β-carboline derivatives against NDV.

(A, B) DF-1 cells were infected with F48E9 (MOI = 0.01). After 1 h adsorption, cells were covered with DMEM containing different concentrations of each compound. At 24 h post-infection, the virus yield in the supernatant was measured by plaque assay (A). The relative mRNA expression was measured by RT-qPCR (B).

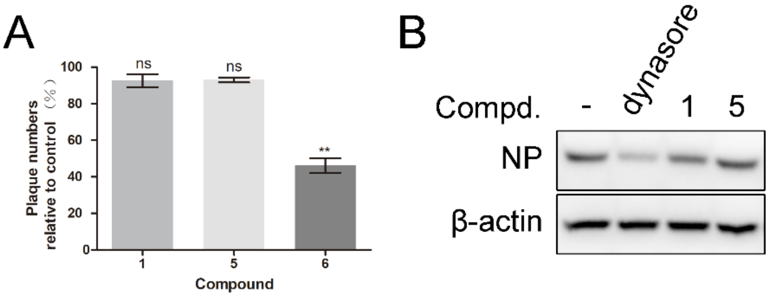

Figure S3. Compound 1 and 5 did not inhibit NDV adsorption and entry.
(A) DF-1 cells were cooled at 4 ℃ for 1 h prior to infection with pre-treatment-NDV at 4 ℃ for 1 h (pre-treatment of NDV: the corresponding compound (20 μM) was added into NDV with titer at 200,000 PFU / 100 μL). Next, this mixture was incubated at room temperature for 2 h before diluting to 100 PFU / 100 μL. Unbound viruses and residual compounds were washed three times with pre-cooled PBS. Next, DF-1 cells were covered with medium-containing methylcellulose (1%). Plaques were visualized and counted by staining them with crystal violet after 72 h.
(B) DF-1 cells were infected with F48E9 (MOI = 5) at 4 ℃ for 1 h. Unbound viruses were washed away by pre-chilled PBS. Next, cells were supplemented with different medium-containing compounds (dynasore: 100 μM; compounds **1**/**5**: 20 μM). At 4 h post-infection, the viral protein was measured by western blot.

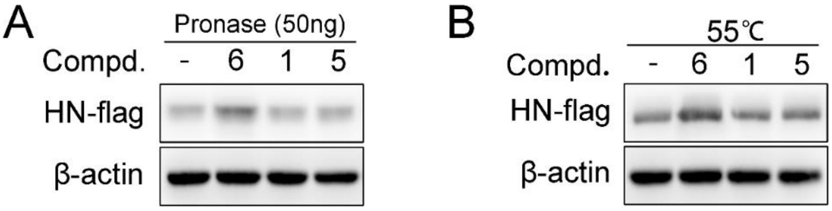

Figure S4. Compound 1 and 5 had no effect on HN stability.
DF-1 cells, transfected with the HN-flag, were lysed with M-PER lysis buffer and incubated with DMSO or indicated compound **1** or **5** (200 μM).
(A) The cell lysates were treated with the pronase (50 ng) and protein was measured by western blot.
(B) The cell lysates were heated at 55 ℃ and protein was measured by western blot.

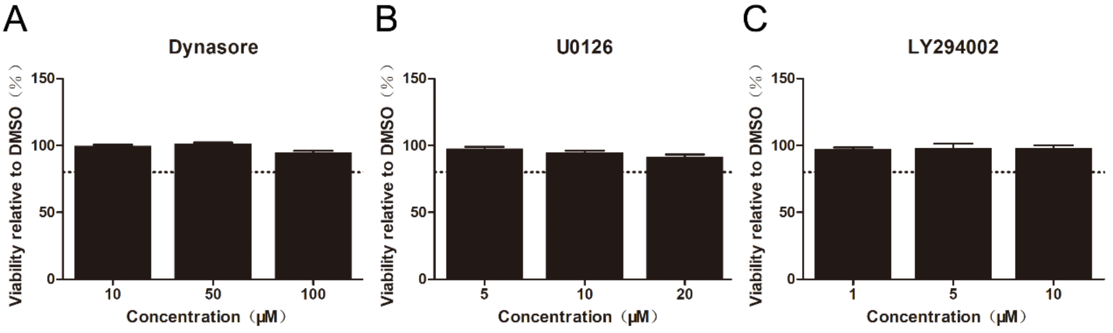

Figure S5. Cell viability of DF-1 cells treated with inhibitors.

(A-C) DF-1 cells were incubated with different concentrations of each inhibitor (A: dynasore; B: U0126; C: LY294002). At 72 h post-incubation, the cell viability was determined using a CCK-8 kit.

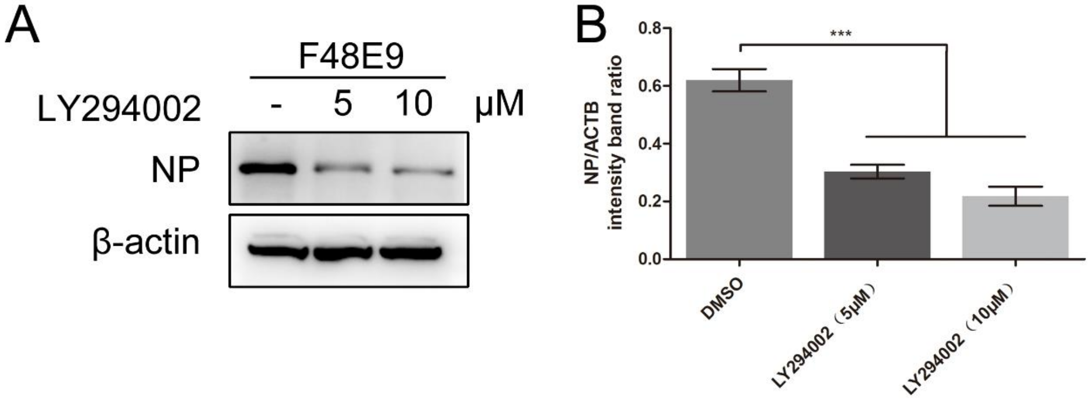

Figure S6. LY294002 inhibits NDV entry into BHK-21 cells.

(A, B) BHK-21 cells were infected with F48E9 (MOI = 5) at 4 °C for 1 h. Unbound viruses were washed away by pre-chilled PBS. Next, cells were supplemented with different medium-containing LY294002. At 4 h post-infection, the viral protein was measured by western blot (A). The targeted protein levels relative to ACTB levels were determined by densitometry (B).
